# *N*-Glycosylation of ß4 Integrin Controls the Adhesion and Motility of Keratinocytes

**DOI:** 10.1371/journal.pone.0027084

**Published:** 2011-11-02

**Authors:** Yoshinobu Kariya, Jianguo Gu

**Affiliations:** 1 Division of Regulatory Glycobiology, Institute of Molecular Biomembrane and Glycobiology, Tohoku Pharmaceutical University, Sendai City, Miyagi, Japan; 2 Department of Biochemistry, Fukushima Medical University School of Medicine, Fukushima City, Fukushima, Japan; Johns Hopkins University, United States of America

## Abstract

α6ß4 integrin is an essential component of hemidesmosomes and modulates cell migration in wound healing and cancer invasion. To elucidate the role of *N*-glycosylation on ß4 integrin, we investigated keratinocyte adhesion and migration through the re-expression of wild-type or *N*-glycosylation-defective ß4 integrin (ΔNß4) in ß4 integrin null keratinocytes. *N*-glycosylation of ß4 integrin was not essential for the heterodimer formation of ß4 integrin with α6 integrin and its expression on a cell surface, but *N*-glycosylation was required for integrin-mediated cell adhesion and migration. Concomitantly with the reduction of ß4 integrin in the membrane microdomain, the intracellular signals of Akt and ERK activation were decreased in cells expressing ΔNß4 integrin. Forced cross-linking of ß4 integrin rescued the decreased ERK activation in ΔNß4 integrin-expressing cells to a similar extent in wild-type ß4 integrin-expressing cells. Surprisingly, compared with cells expressing wild-type ß4 integrin, an alternation in *N*-glycan structures expressed on epidermal growth factor receptor (EGFR), and the induction of a stronger association between EGFR and ß4 integrin were observed in ΔNß4 integrin-expressing cells. These results clearly demonstrated that *N*-glycosylation on ß4 integrin plays an essential role in keratinocyte cellular function by allowing the appropriate complex formation on cell surfaces.

## Introduction

α6ß4 integrin is expressed primarily in basal epithelial cells and acts as a receptor for the laminins of the basement membrane [1]. In the skin, α6ß4 integrin plays a role in the maintenance of epidermal integrity through formation of the hemidesmosome complex, which serves as an anchor for basal keratinocytes to the underlying basement membrane through its association with laminin-332 (previously called laminin-5) [1], [2]. In fact, mutations in the ß4 integrin subunit cause a skin blistering disease called pyloric atresia that is associated with junctional epidermolysis bullosa [1], [3]. In contrast to its function in stable adhesion, α6ß4 integrin also plays a key role in keratinocyte migration during wound healing and cancer cell invasion [4], [5], [6], [7]. The α6ß4 integrin combines with several receptor tyrosine kinases (RTKs) such as epidermal growth factor receptor (EGFR), ErbB2 and c-Met [4], [8]. Upon growth factor stimulation, these RTKs activate Src family kinase (SFK), and thereby phosphorylate the cytoplasmic domain of ß4 integrin. The α6ß4 integrin signaling proceeds through SFK-mediated phosphorylation of the unique cytoplasmic domain of ß4 and activation of PKC from PI3K to Akt [9] and Ras to ERK [10]. Conversely, α6ß4 integrin promotes the SFK-dependent phosphorylation of RTKs. The palmitoylation of α6ß4 integrin promotes lipid rafts incorporation and SFK association [11]. This signaling causes a disruption in hemidesmosomes and an increase in keratinocyte cell motility [4].

Most receptors on the cell surface, along with secreted proteins, are *N*-glycosylated. *N*-glycosylation has a profound effect on protein folding, stability, and protein-protein interactions [12]. The presence of *N*-glycans on α5ß1 integrin, which is the best-characterized integrin molecule, reportedly is required for αß heterodimer formation and proper integrin-extracellular matrix (ECM) interactions [13], [14]. Furthermore, changes in the *N*-glycan structures of integrins can affect cell-cell adhesion and cell-ECM interactions, thereby affecting cell adhesion and migration. In one of our recent reports, galectin-3 bound to α6ß4 integrin and the EGFR complex, and its formation was partially inhibited by lactose treatment, suggesting that the association of α6ß4 integrin with EGFR was partially dependent on galectin-3-mediated cross-linking through their *N*-glycans [15]. This might indicate that *N*-glycans on α6ß4 integrin could affect an association with other molecules, thereby changing the related signaling and cellular function. Until recently, however, no report had described *N*-glycosylation on α6ß4 integrin and its functional significance. Here, we report that α6ß4 integrin can be modified by *N*-glycosylation, and *N*-glycans on ß4 integrin regulates keratinocyte cell adhesion and migration by affecting the association of α6ß4 integrin with other molecules such as laminin-332 and EGFR.

## Results

### Integrin ß4 subunit undergoes *N*-glycosylation in keratinocyte cells

**Figure 1:**
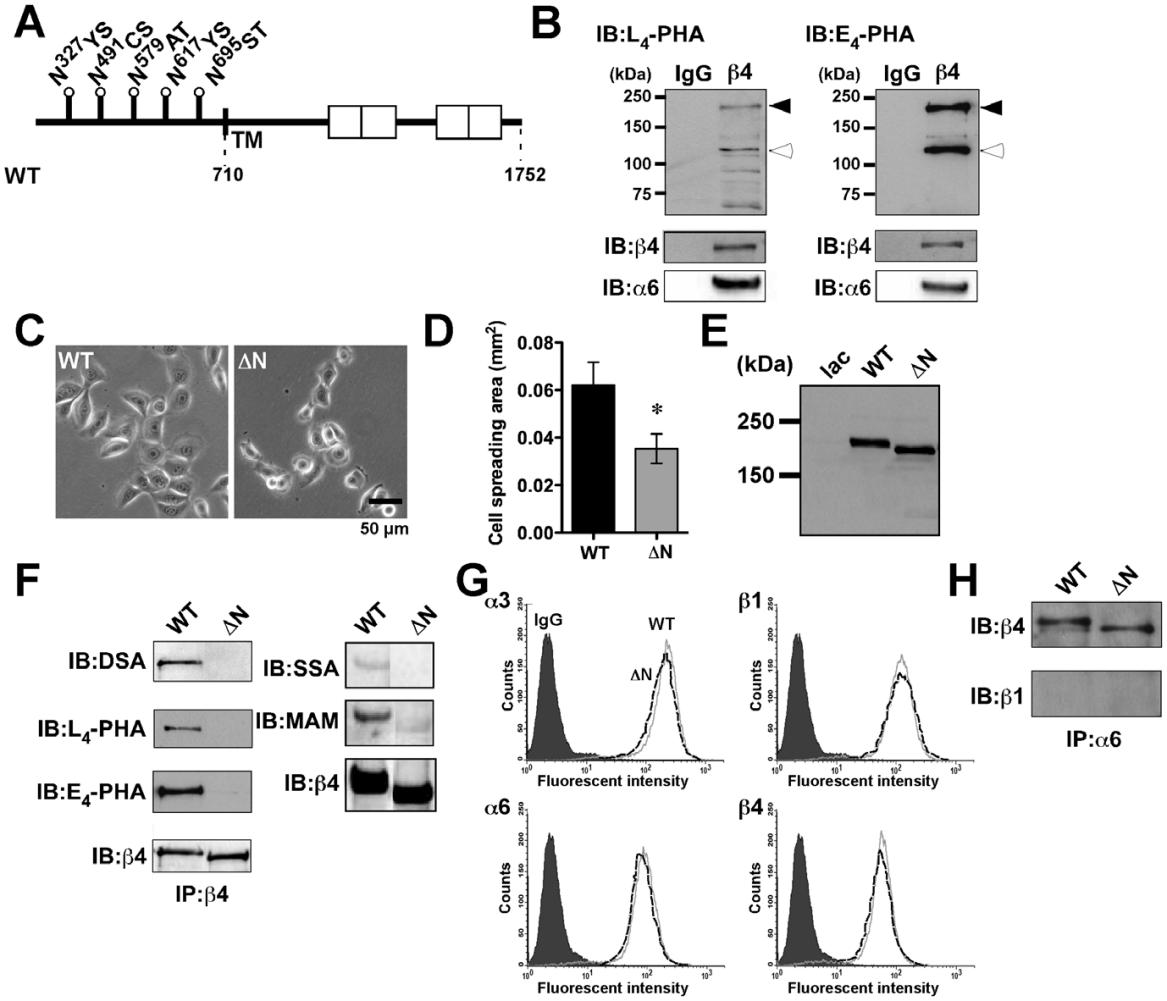
Characterization of *N*-glycosylation-defective ß4 integrin-expressing keratinocytes. (A) Schematic diagram of the potential *N*-glycosylation sites (N*X*(S/T)) on the ß4 integrin subunit. The sites corresponding to the putative *N*-glycosylation sites on the ß4 integrin subunit (Asn^327^, Asn^491^, Asn^579^, Asn^617^, and Asn^695^) are shown by flags. Numbers and boxes indicate the number of amino acid residue and the four intracellular fibronectin type III repeats, respectively. TM, transmembrane region. (B) Cell lysates from normal keratinocytes were immunoprecipitated using a control IgG or an anti-ß4 integrin Ab. Immunoprecipitates were run on a 6% SDS-polyacrylamide gel and probed with the biotinylated L_4_-PHA lectin (upper left panel) and E_4_-PHA lectin (upper right panel) or anti-ß4 and anti-α6 integrin Abs (lower panels). IB, immunoblot. The black and white arrowheads indicate the ß4 integrin and α6 integrin subunits, respectively. Ordinates indicate molecular sizes in kDa of marker proteins. (C) Cell morphology of the WT and ΔNß4 integrin-expressing keratinocytes during cell culture. (D) The cell-spreading area in A was calculated using computer software (Image J). Each bar represents the mean ± S.D. of triplicate assays. *p<0.001 (unpaired t-test vs.WT). (E) 20 µg of cell lysates from control lacZ- (lac), WTß4, ΔNß4 integrin-expressing keratinocytes were run on a 6% gel under reducing conditions, blotted onto a nitrocellulose membrane, and then probed with an anti-ß4 integrin Ab. (F) Cell lysates were immunoprecipitated using a polyclonal Ab against ß4 integrin. Immunoprecipitates were run on a 6% SDS-polyacrylamide gel and probed with the indicated biotinylated lectins or an anti-ß4 integrin Ab. (G) Cell surface expression levels of α3, α6, ß1 and ß4 integrin subunits of WT or ΔNß4 integrin-expressing keratinocytes were examined using FACS analysis. Prior to analysis, cells were incubated with either the indicated integrin Abs or control IgG, followed by incubation with Alexa Fluor conjugated secondary Abs, as described under “MATERIALS AND METHODS.” (H) Immunoprecipitates from WT or ΔN keratinocytes with an anti-α6 integrin were probed with an anti-ß4 (upper panel) or ß1 integrin (lower panel) Ab.

ß4 integrin has five potential *N*-glycosylation sites, Asn^327^, Asn^491^, Asn^579^, Asn^617^ and Asn^695^, on its molecule (Figure 1A). One of our previous reports showed that the ß4 integrin-EGFR complex in keratinocytes is partially inhibited by lactose treatment, suggesting that ß4 integrin can undergo *N*-glycosylation [15]. To examine this possibility, the *N*-glycosylation state of ß4 integrin in normal keratinocytes was investigated by immunoprecipitation using ß4 integrin antibody (Ab) and lectin blotting. Both L_4_-PHA (leukoagglutinating phytohemagglutinin) and E_4_-PHA (erythroagglutinating phytohemagglutinin) lectin, which recognize ß1,6GlcNAc and bisecting GlcNAc, respectively, reacted with ß4 integrin, suggesting that *N*-glycosylation could occur in a ß4 integrin molecule (Figure 1B). In addition to that, the results showed that α6 integrin could also be *N*-glycosylated (Figure 1B). To investigate which potential sites are actually occupied by *N*-glycans, five single potential *N*-glycosylation site-defective mutants (ΔN^327^, ΔN^491^, ΔN^579^, ΔN^617^, and ΔN^695^), which replaced the Asparagine (N) to Glutamine (Q) conversion on each of five potential *N*-glycosylation sites (N*X*(S/T)) on ß4 integrin, and three mutant forms with a combination of these mutation sites (ΔN^327, 491, 579^, ΔN^617, 695^, ΔN^579, 617, 695^) or wild-type (WT) ß4 integrin retrovirus expression vectors, were prepared. Then the cDNA constructs were independently introduced into the ß4 null keratinocytes obtained from a patient with epidermolysis bullosa accompanied by pyloric atresia (ΔN^327^, ΔN^491^, ΔN^579^, ΔN^617^, ΔN^695^, ΔN^327, 491, 579^, ΔN^617, 695^, ΔN^579, 617, 695^ or WT keratinocytes). The immunoblotting analysis revealed that *N*-glycosylation occurred in at least four potential sites, Asn^327^, Asn^491^, Asn^579^, and Asn^695^, since the band migratory on SDS-PAGE of these mutants was faster than that of WT (Figure S1A and S1C). On the other hand, it was difficult to judge whether Asn^617^ was occupied by *N*-glycans since the band mobility of the mutant is almost the same as that of the WT. When the cell morphology of those mutants were compared, it was difficult to detect the effect of each single mutation or a combination of these mutations on cell spreading compared with that of WT keratinocytes (Figure S1B and S1D). These results suggest that all of *N*-glycosylation sites on ß4 integrin are important for cell spreading.

To further investigate the significance of *N*-glycosylation on ß4 integrin, *N*-glycosylation-defective ß4 integrin mutant (ΔN) retrovirus expression vector, which replaced the Asparagine (N) to Glutamine (Q) conversion on all five potential *N*-glycosylation sites on ß4 integrin, was also prepared and infected into the ß4 null keratinocytes. Finally, ΔNß4 integrin expressing ß4 null keratinocytes (ΔN keratinocytes) was established after a week selection with blasticidine S. During cell culture, cell spreading had decreased more for ΔN keratinocytes than for WT keratinocytes (Figures 1C and 1D). Because α6ß4 integrin uses laminin-332 as a major substrate, it is possible that the inhibition of cell spreading of ΔN keratinocytes was caused by aberrant laminin-332 secretion and deposition. However, the secretion (Figure S2A, CM) and deposition (Figure S2A, ECM and S2B) of laminin-332 and hemidesmosome formation (Figure S2C) were quite normal for both ΔN keratinocytes and WT keratinocytes. Therefore, the decreased cell spreading of ΔN keratinocytes was likely caused by an impaired association between ß4 integrin and laminin-332 rather than by changes in laminin-332 secretion and deposition.

The expression of ΔNß4 integrin was confirmed by immunoblotting using a ß4 integrin Ab and the cell lysates of ΔN keratinocytes. The band of ΔNß4 integrin migrated faster than that of WT ß4 integrin in SDS-PAGE gel, the predicted cause of which was a lack of *N*-glycosylation whereas control lacZ expressing ß4 integrin null keratinocytes (lac keratinocytes) showed no band (Figure 1E). To further check the *N*-glycosylation state of ΔNß4 integrin, lectin blotting using DSA (*Datura stramonium* agglutinin), L_4_-PHA and E_4_-PHA lectins, which preferentially bind to branched sugar chains (more than triantennary), ß1,6-branched GlcNAc residues and bisecting GlcNAc residues in *N*-glycans, respectively, was done against ΔNß4 integrin as well as WT ß4 integrin. As a result, WT ß4 integrin, but not ΔNß4 integrin, was detected in all those lectins (Figure 1F, left panel). Interestingly, the staining of SSA (*Sambucus sieboldiana* agglutinin) and MAM (*Maackia amurens* mutagen) lectins, which recognize α2–6 and α2–3 sialyl linkages, respectively, showed a decrease, but not a deficiency, in the sialylation levels in ΔNß4 integrin compared with that in WT ß4 integrin (Figure 1F, right panel), which strongly supported the presence of *O*-glycan(s) in ß4 [16]. In general, the *N*-glycosylation of secreted and transmembrane proteins is important for their secretion and cell surface expression. Therefore, it might be impossible for ΔNß4 integrin to express on a cell surface. To exclude the possibility, cell surface expression of ß4 integrin in WT and ΔN keratinocytes was checked by FACS analysis. As a result, there were no differences in expression levels of ß4, or in ß1, α3 and α6 integrins (Figure 1G) on the surface between the two cells. These data suggest that *N*-glycosylation on ß4 integrin is not essential for its expression on a cell surface. α6 integrin is known to be expressed as an α6ß4, rather than anα6ß1, integrin in keratinocytes [2]. To examine whether the loss of *N*-glycosylation on ß4 integrin affects its heterodimeric formation with α6 integrin, the immunoprecipitates of α6 were immunoblotted with anti-ß1 and ß4 integrin Abs. As shown in Figure 1H, α6ß4 integrin heterodimer, but not α6ß1 integrin, was detected in both WT and ΔN keratinocyte cell lysates, suggesting that *N*-glycosylation on ß4 integrin does not affect the complex formation with α6 integrin in keratinocytes. Considering the results of the ΔN keratinocytes including the cell-spreading assay, it seems that ΔNß4 integrin but not single *N*-glycosylation mutants and mutant forms with a combination of two or three mutation sites would be a good model to demonstrate the significance of *N*-glycosylation on ß4 integrin in keratinocyte function, because the effect of ΔNß4 integrin on keratinocyte function was apparent. Therefore, we decided to examine the effect of *N*-glycosylation on ß4 integrin on cell function using WT and ΔN keratinocytes.

### *N*-glycosylation-defective ß4 integrin decreases keratinocyte cell adhesion and migration on laminin-332

**Figure 2:**
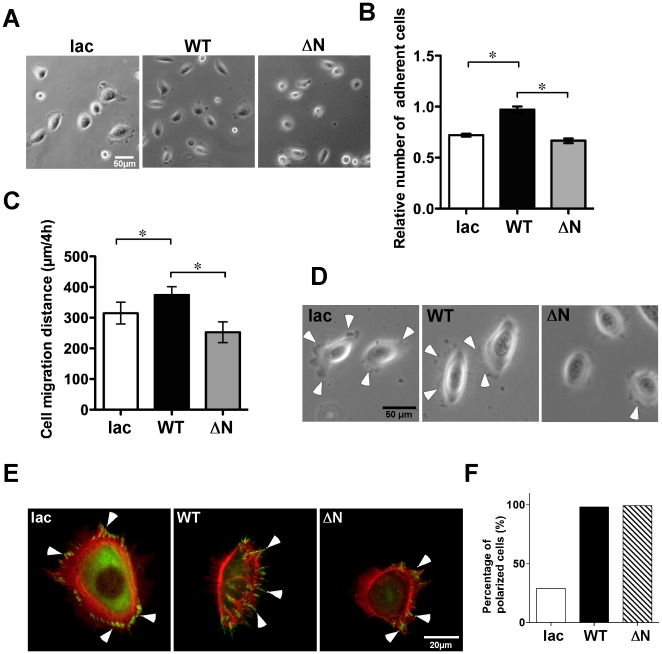
Effects of ΔNß4 integrin on the cellular activities on laminin-332 substrate. (A) Cell morphology of the indicated keratinocytes was photographed after cell spreading on laminin-332 substrate for 20 min. (B) The effect of ΔNß4 integrin on cell adhesion activity on 2 µg/ml of the laminin-332 substrate. The experiment was performed in triplicate thrice. *P<0.001 (one-way ANOVA, Bonferroni post test) vs. WT. (C) Effect of ΔNß4 integrin on the cell migration activity on laminin-332 substrate. The indicated keratinocytes in culture medium were plated onto dishes precoated with 2 µg/ml laminin-332, followed by incubation for 1 h. Cell migration was monitored by time-lapse microscopy, as described in “MATERIALS AND METHODS.” Each bar represents the mean ± S.D. of the migration distance of ten cells in each assay in three independent experiments. *P<0.001 (one way ANOVA, Bonferroni post test) vs. WT. (D) Cell morphology of each keratinocyte during migration. (E) The indicated keratinocytes in culture medium were plated onto dishes precoated with 2 µg/ml laminin-332. After incubation for 24 h, cells were fixed and stained with a paxillin mAb and followed by secondary Ab and phalloidin for visualizing paxillin (green) and actin filament (red). (F) Polarized cells were determined and counted as described in “MATERIALS AND METHODS.”

α6ß4 integrin promotes cell adhesion and migration through its association with laminin-332 [6]. When WT and ΔN keratinocytes were plated on laminin-332 coated plates, cell spreading and adhesion were decreased more in ΔN keratinocytes when compared with WT keratinocytes (Figures 2A and 2B). To investigate the effect of a lack of *N*-glycosylation on ß4 integrin on keratinocyte cell motility on laminin-332, WT and ΔN keratinocyte cell motility was assayed using a time-lapse microscopy system. The cell motility of ΔN keratinocytes was significantly decreased when compared with WT keratinocytes (Figure 2C). It was apparent that the lamellipodia formation was consistently inhibited in ΔN keratinocytes (Figure 2D). On the other hand, the cell spreading of lac keratinocytes was comparable to that of WT keratinocytes (Figure 2A). However, the cell adhesion and migration of lac keratinocytes significantly decreased when compared with WT keratinocytes (Figures 2B and 2C). During cell migration, focal contacts that included paxillin were formed at the front of moving cells generating a motile force that determined the cell motile polarity and direction. ß4 integrin reportedly is a critical molecule for determining cell motile polarity and direction during keratinocyte cell migration [6], [17]. To examine the effects of *N*-glycosylation on the cell polarity of ß4 integrin, cells spread on laminin-332 were stained with anti-paxillin Ab. The lac keratinocytes showed lamellipodia and paxillin staining in various directions whereas paxillin staining was only observed at the cell front in both WT and ΔN keratinocytes (Figures 2D and 2E). Coincident with the results of paxillin staining, the percentage of polarized cells in both WT and ΔN keratinocytes was almost 100%, whereas that in the control cells (lac) was 29% (Figure 2F). Taken together, *N*-glycosylation of ß4 integrin affected the keratinocyte cell motility by changing the cell adhesion and lamellipodia formation, but it did not affect cell motile polarity.

### Lack of *N*-glycosylation on ß4 integrin suppresses localization of ß4 integrin in caveolae, and PI3K and ERK signaling

**Figure 3:**
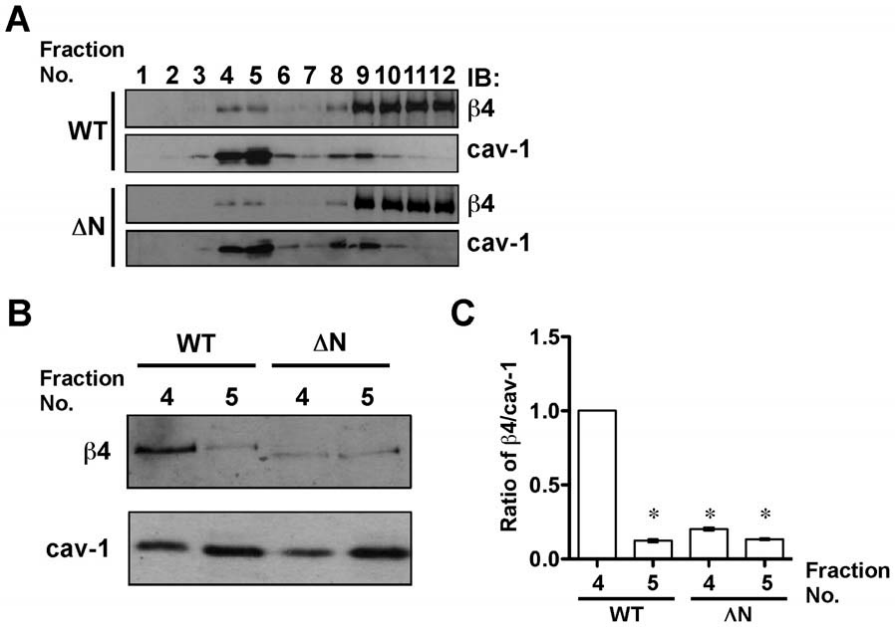
Effects of ΔNß4 integrin on lipid raft localization. (A) After lysis in 1% Brij98, WT or ΔNß4-expressing keratinocytes were fractionated by sucrose gradient ultracentrifugation. The fractions from each WT and ΔN keratinocytes were independently blotted to the membrane and probed with antibody against ß4 integrin (ß4), and then reprobed with antibody against caveolin-1 (cav-1). (B) Fractions 4 and 5, which were lipid raft fractions, were blotted onto the same membrane and probed with antibody to ß4 integrin (ß4), and then reprobed with antibody to caveolin-1 (cav-1). (C) Results of the densitometric analysis are shown as the integrated density of the ratio of ß4 integrin to caveolin-1 bands in Figure 3B, which was 1.0 for fraction 4 from WT keratinocytes. *P<0.001 (one-way ANOVA, Bonferroni post test) vs. WT. All blots are representative for at least three independent experiments.

α6ß4 integrin can localize in the caveolae where signaling molecules accumulate to transduce cellular signaling. In fact, the α6ß4 palmitoylation mutant, which cannot localize in the caveolae, decreased some ß4 integrin-inducing signaling [11], [18]. To examine whether the decreased functional activities such as cell adhesion and migration in ΔN keratinocytes is related to the localization of ß4 integrin in the caveolae, we checked localization into the caveolae of both ΔNß4 integrin and WT ß4 integrin using a sucrose gradient density method [11]. The rafts were recovered from fractions 4 and 5, as represented by the relative enrichment of caveolin-1 in both WT and ΔN keratinocytes (Figure 3A). Equal aliquots from each keratinocyte were independently blotted with anti-ß4 integrin Ab, suggesting that both WT ß4 and ΔNß4 integrin cofractionated with the rafts (Figure 3A). To more precisely examine the effect of *N*-glycan on ß4 integrin on raft localization of ß4 integrin, the samples from fractions 4 and 5 were transferred to the same membrane and immunoblotted with anti-ß4 Ab and then reprobed with anti-caveolin-1 Ab (Figure 3B). As a result, WT ß4 integrin was efficiently localized in fraction 4 compared with ΔNß4 integrin because the ratio of ß4 integrin to caveolin-1 in fraction 4 from WT keratinocytes was higher than that from ΔN keratinocytes (Figures 3B and 3C). These results suggest that *N*-glycosylation on ß4 integrin is required for the localization of ß4 integrin in the caveolae, as well as for downstream signaling.

**Figure 4:**
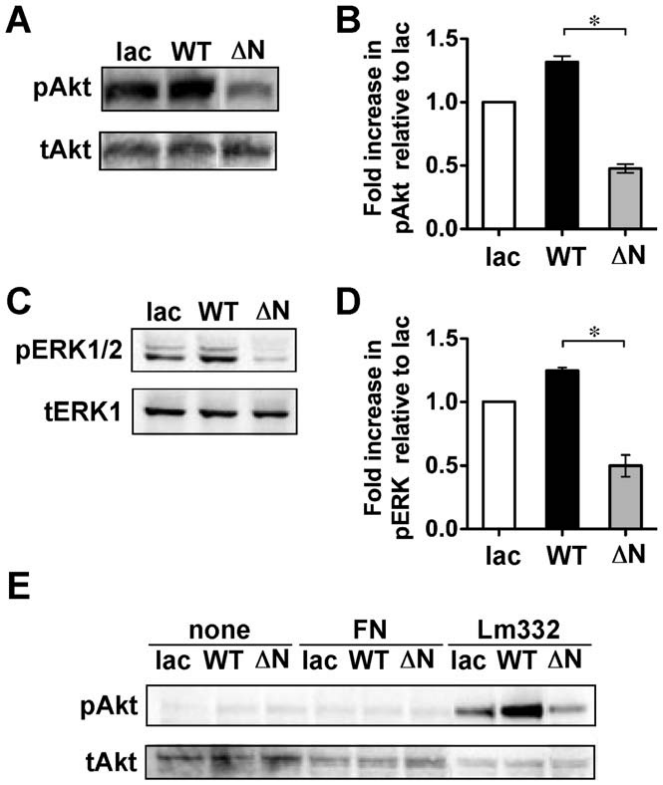
Effects of ΔNß4 integrin on cellular signaling. Lac, WT or ΔN keratinocytes were grown to semi-confluence and then each cell lysate was collected as described in “MATERIALS AND METHODS. ” (A and C) 20 µg of cell lysates from those keratinocytes were run on 7.5% or 5–20% SDS-polyacrylamide gel and probed with a phospho-Akt Ab (A, upper panel) or phospho-ERK1/2 Ab (C, upper panel), and then reprobed with a total Akt Ab (A, lower panel) or total ERK Ab (C, lower panel), respectively. (B and D) Results of the densitometric analysis are shown as the integrated density of the ratio of phospho-protein to total protein bands, which was 1.0 for lac keratinocytes. *p<0.001 (one-way ANOVA, Bonferroni post test for WT) vs. ΔN. (E) Cells in supplement-free media were plated to the indicated substrates. After 30 minutes incubation, cell lysates were collected and immunoblotted against the indicated antibodies. All blots are representative of at least three independent experiments: none, plastic; FN, fibronectin; and, Lm332, laminin-332.

α6ß4 integrin is known to upregulate both PI3K and ERK signaling, which promotes cell adhesion and migration, through an association with laminin-332 [4]. When WT and ΔN keratinocytes were plated on laminin-332, phosphorylation levels of both Akt (Figures 4A and 4B) and ERK (Figures 4C and 4D) in ΔN keratinocytes were downregulated to less than half the levels found in WT keratinocytes. These results agree with the observation that cell adhesion and migration of ΔN keratinocytes was suppressed on a laminin-332 substrate (Figures 2B and 2C). To examine whether the cellular signaling was actually activated by laminin-332, we compared the phosphorylation levels of Akt when keratinocytes were plated on control plastic, fibronectin and laminin-332 (Figure 4E). The phosphorylation signals of Akt were detected only when keratinocytes were plated on laminin-332, suggesting that the Akt activation in keratinocytes mainly laminin-type (i.e. α3ß1 and α6ß4) integrin-mediated signaling. Intriguingly, lac keratinocytes on laminin-332 showed higher levels of phosphorylated Akt (Figures 4A, 4B and 4E) and ERK (Figures 4C and 4D) than those in ΔN keratinocytes on laminin-332, implicating integrin-mediated cellular signaling might be different between ß4-deficient and ß4-expressing keratinocytes.

**Figure 5:**
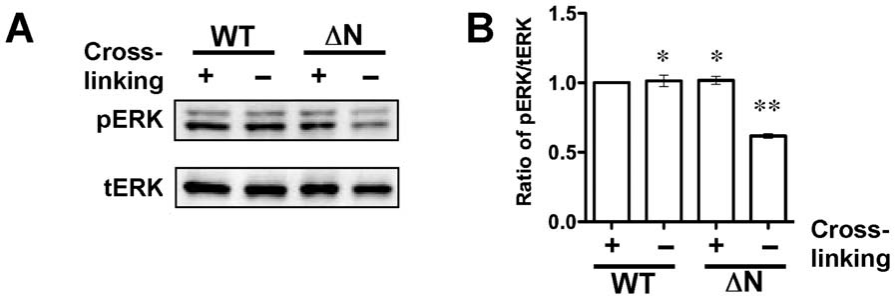
Effect of ß4 integrin Ab-mediated α6ß4 integrin clustering on phosphorylation of ERK. (A) WT and ΔN keratinocytes were incubated on ice for 30 min in the presence of rat anti-ß4 integrin mAb. The cells were washed and then incubated in the presence (cross-linking, +) or absence (cross-linking, –) of a rabbit anti-rat secondary Ab at 37°C for 10 min. Following incubation, the cells were analyzed for the phosphorylation of ERK by immunoblotting. (B) Results of the densitometric analysis are shown as the integrated density of the ratio of phospho-ERK (pERK) to total ERK (tERK) bands, and the ratio in WT keratinocytes with cross-linking (WT, +) was 1.0. *P>0.05, **p<0.001 (one-way ANOVA, Bonferroni post test) vs. WT with cross-linking. All blots are representative of at least three independent experiments.

Since the *N*-glycosylation-mediated supramolecular complex formation induces α6ß4 integrin clustering, and the resultant ERK phosphorylation [15], it might be possible that the aberrant *N*-glycosylation on the ß4 integrin impaired proper integrin clustering. To check this hypothesis, clustering assay was done using an anti-ß4 integrin Ab and the secondary Ab. The assay that had shown decreased phosphorylation levels of ERK in ΔN keratinocytes was greatly increased to a similar level found in WT keratinocytes by cross-linking in the presence of the secondary Ab (Figures 5A and 5B). These results suggest that *N*-glycosylation on the ß4 integrin plays important roles in proper integrin clustering and the following cellular signaling.

### Lack of *N*-glycosylation on ß4 integrin affects *N*-glycan on EGFR and association between ß4 integrin and EGFR

**Figure 6:**
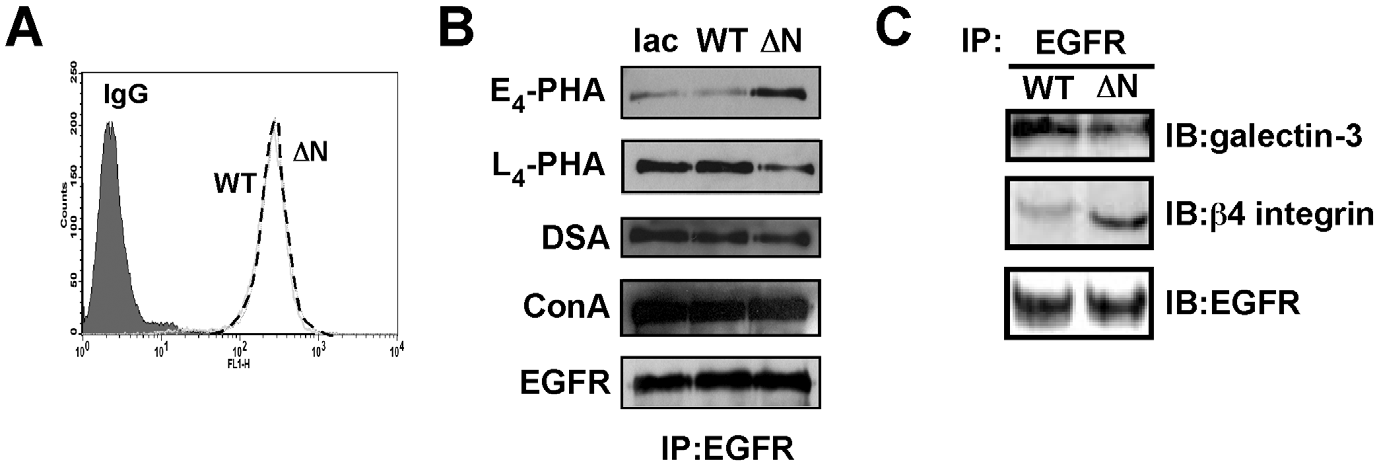
*N*-glycosylation of ß4 integrin affects the *N*-glycosylation state of EGFR and galectin-3 mediated ß4 integrin-EGFR complex formation. (A) Cell surface expression levels of EGFR by FACS analysis against WT and ΔN keratinocytes. (B) Cell lysates from lac, WT and ΔN keratinocytes were immunoprecipitated using an anti-EGFR pAb. Immunoprecipitates were run on a 6% SDS-polyacrylamide gel and were probed with either the biotinylated E_4_-PHA lectin, L_4_-PHA lectin, DSA lectin and ConA lectin or an anti-EGFR Ab. (C) Covalent cross-linking was performed on WT and ΔN keratinocytes. After cross-linking, collected cell lysates were immunoprecipitated using an anti-EGFR pAb. Immunoprecipitates were run on a 5–20% SDS-polyacrylamide gel and were probed with the indicated Abs.

Our previous reports showed that the association of ß4 integrin with EGFR was partially inhibited by lactose, which is a competitive inhibitor of galectin binding to ß-galactoside residue [15]. In addition, the association through *N*-glycans induced ß4 integrin clustering, which resulted in ERK phosphorylation [15]. These results may suggest that *N*-glycans on ß4 integrin play a pivotal role in the association of ß4 integrin with EGFR and their downstream signaling. To clarify this possibility, we tried to examine the ß4 integrin-EGFR complex in both WT and ΔNß4 integrin-expressing keratinocytes after cross-linker treatment. First, the expression levels of EGFR on the cell surface in ΔN and WT keratinocytes were analyzed by flow cytometry using anti-EGFR monoclonal Ab (mAb). As a result, there was no difference in EGFR expression on the cell surface between the two cells (Figure 6A). Next, to examine whether the defect of *N*-glycosylation on ß4 integrin influences *N*-glycosylation of EGFR, the *N*-glycosylation state of EGFR for lac, ΔN and WT keratinocytes were analyzed by lectin blotting using E_4_-PHA, L_4_-PHA, DSA and ConA (Concanavalin A) lectins. Surprisingly, EGFR in ΔN keratinocytes increased E_4_-PHA reactivity but decreased L_4_-PHA and DSA reactivity compared with that in lac and WT keratinocytes, although the results of ConA blotting were the same in all 3 keratinocytes (Figure 6B). These results indicated that the expression of *N*-glycans containing bisecting GlcNAc residues on EGFR was increased whereas the expression of ß1,6 GlcNAc-containing *N*-glycans was decreased in ΔN keratinocytes. Since it is easier for the poly-*N*-acetyllactosamine sequence, which is a preferred ligand for galectin, to elongate the ß1,6GlcNAc branch, it is possible that the binding ability of galectin-3 to EGFR in ΔN keratinocytes was decreased when compared with that in WT keratinocytes. This would have resulted in a reduction of the galectin-3 mediated ß4 integrin-EGFR complex.

To examine that possibility, galectin-3 mediated ß4 integrin-EGFR complex formation was assessed by immunoprecipitation using EGFR polyclonal Ab (pAb) after cross-linker treatment. The co-immunoprecipitates of galectin-3 using anti-EGFR pAb revealed that galectn-3 binding to the ß4 integrin-EGFR complex was decreased in ΔN keratinocytes when compared with WT keratinocytes (Figure 6C, upper panel). Unexpectedly, the bound ß4 integrin to EGFR in the ß4 integrin-EGFR complex was increased more in ΔN keratinocytes than in WT keratinocytes (Figure 6C, middle panel), indicating that *N*-glycans of ß4 integrin may negatively regulate the complex formation.

## Discussion

**Figure 7:**
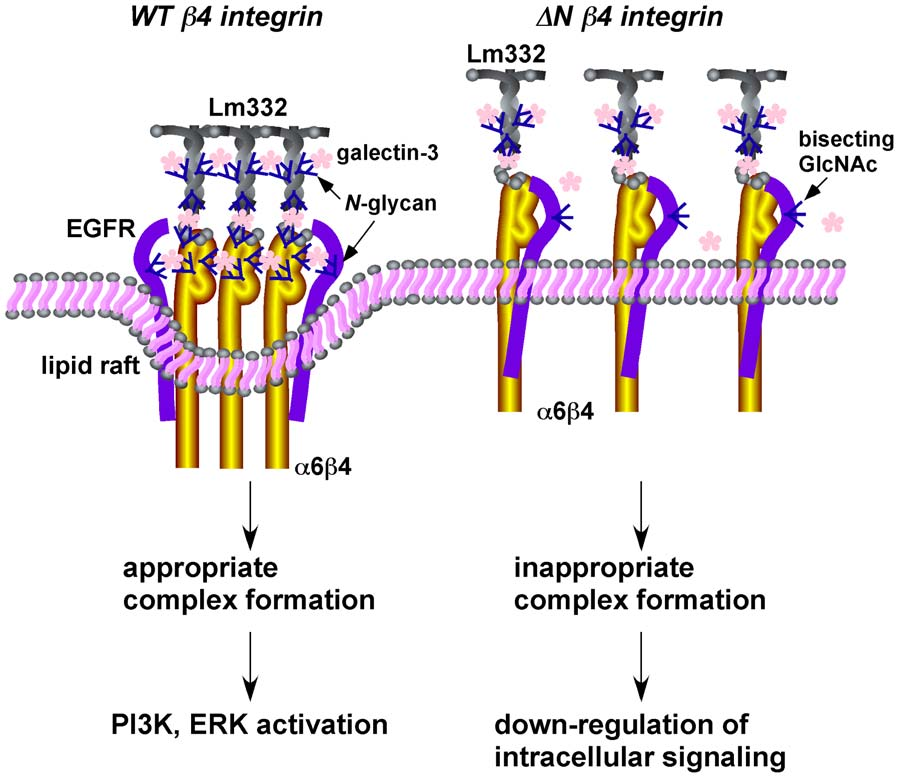
Hypothetical model for the effect of *N*-glycans on ß4 integrin cellular function and signal transduction. Under normal conditions (WT ß4 integrin), ß4 integrin binds to EGFR and laminin-332 (Lm332) through cross-linking with galectin-3-mediated *N*-glycans, which induces a modest association among them, and thereby efficient integrin clustering, cellular signaling, and cell migration. However, in ΔNß4 integrin-expressing keratinocytes, the lack of galectin-3-meditaed ß4 integrin cross-linking and alternation in *N*-glycosylation of EGFR, resulted in a strong protein-protein association between ß4 integrin and EGFR, and a weak association between ß4 integrin and laminin-332, which inhibited efficient integrin clustering, thereby suppressing cellular signaling and cell migration. The *N*-glycan of ß4 integrin is also important for the lipid raft localization where SFKs-mediated phosphorylation takes place.

In the present study, we constructed an assay system to examine the biological functions of *N*-glycans on ß4 integrin, which included ß4 integrin expression in ß4 integrin-null keratinocyte and ß4 integrin-mediated cell adhesion, migration, and intracellular signaling. An important finding was that *N*-glycan on ß4 integrin not only controlled its association with other molecules such as EGFR, but also modulated its activation and cellular signal transduction, introducing the concept that glycan-mediated soft interactions, which would generate the space between ß4 integrin and its associated molecules, could be more important than the stronger associations by protein-protein interactions (Figure 7).

*N*-glycosylation has a profound effect on protein folding, stability, and cell-surface expression. Indeed, *N*-glycosylation on the α5 or ß1 subunit is indispensable for α5ß1 integrin heterodimer formation and cell-surface expression [13], [14], [19]. Our results, however, clearly demonstrate that the lack of *N*-glycosylation on the ß4 integrin subunit had no effect on α6ß4 heterodimer formation and its cell surface expression. Similarly, deglycosylation of αMß2 integrin altered neither its amount on cell surfaces nor the αMß2 integrin complex formation [20]. In the case of ß1 integrin, three *N*-glycosylation sites, Asn^212^, Asn^269^, and Asn^363^, within the I-like domain could be essential for α5ß1 integrin cell surface expression and heterodimeric formation [19]. The ß1 integrin lacking the specificity-determining loop within the I-like domain, which contains one *N*-glycosylation site, Asn^212^, can form a heterodimer with α4 and α5 integrin but not with α6 and αV integrin [21]. Taken together, these findings might imply that the effects of *N*-glycosylation on integrin on its cell surface expression and heterodimeric formation are integrin subunit-dependent.

Although *N*-glycosylation on ß4 integrin was not required for heterodimeric formation of α6ß4 integrin and cell-surface expression, the removal of *N*-glycosylation on ß4 integrin significantly inhibited α6ß4 integrin-mediated cellular function such as cell spreading, adhesion and migration. Furthermore, ΔNß4 integrin decreased the advantages of localization in a lipid raft, integrin clustering, and ERK phosphorylation. Compartmentalization in lipid rafts is very important for integrin signaling and clustering [22]. In fact, palmitoylation-defective α6ß4 integrin, which diminished incorporation into a lipid raft, did not activate the SFK signaling to ERK [11]. Therefore, cell adhesion to laminin-332 through ß4 integrin could be positively regulated by lipid raft localization. On the other hand, impaired lipid raft localization of ß4 integrin did not affect the association with EGFR [18]. Therefore, it is likely that the cooperative phosphorylation event between ß4 integrin and EGFR, occurs outside the lipid raft. Although it is speculation, since galectin-3 can bind to ß4 integrin through *N*-glycan [15] and has been detected in lipid rafts [23], [24], galectin-3 might play an important role in facilitating ß4 integrin incorporation into a lipid raft. However, because the amount of WT ß4 integrin molecules localized in the raft fraction were small, it is difficult to consider that only lipid raft localization of ß4 integrin could be responsible for the high Akt and ERK phosphorylation levels in WT keratinocytes, as compared to ΔN keratinocytes, after plating on laminin-332. Therefore, there may be other underlying mechanisms by which *N*-glycan on ß4 integrin upregulates ß4 integrin-dependent cellular signaling.

Unexpectedly, ß4 integrin-deficient keratinocytes could spread more efficiently than ΔN keratinocytes, as shown in Figure 2A, although cell adhesion and migration on laminin-332 was comparable (Figures 2B and 2C). ß4 integrin-deficient keratinocytes express an α3ß1 integrin, which is another laminin-332 receptor. As many groups have previously reported [25], [26], [27], [28], α6ß4 integrin affects α3ß1 integrin function in cell spreading and migration. Therefore, the effects of laminin-332 on cell functions in ß4 integrin-deficient keratinocytes might have opened an alternative pathway that depends on α3ß1 integrin.

The deletion of all 5 potential *N*-glycosylation sites on ß4 integrin promoted the EGFR-ß4 integrin association (Figure 6C). Similarly, tetraspanin CD82 with incomplete *N*-glycosylation exhibits an enhanced association with the α3 and α5 integrin subunits [29]. Therefore, *N*-glycans which would generate the space between two molecules may have a suppressive effect on protein-protein interaction *in vivo* [12]. Since sialylation plays important roles in intermolecular interactions either by its negative charge or by the sialic acid binding of Ig-like lectins [30], the enhanced associations between ΔNß4 integrin and EGFR, could be also explained by the significant decrease of sialic acid on ß4 integrin (Figure 1F). A modest association between laminin-332 and α6ß4 integrin mediated by galectin-3 through the *N*-glycans on both molecules promoted cell adhesion and migration on laminin-332 as well as α6ß4 integrin clustering on laminin-332 [15], [31] (Figure 7). Accordingly, an appropriate intermolecular interaction through *N*-glycan is important for the efficient cellular signaling and the following cellular function. Deletion of *N*-glycans on ß4 integrin caused the decreased ß1,6GlcNAc residues but increased bisecting GlcNAc residues on EGFR molecule (Figure 6B) although details of the molecular mechanism in this novel result will require further studies. As with our previous results [31], increased bisecting GlcNAc residue seems to suppress further addition of ß1,6GlcNAc residue, which is a preferred ligand for galectin-3, on EGFR molecule. This decreased ß1,6GlcNAc residue on EGFR could also affect on galectin-3 dependent association between EGFR-ß4 integrin complex formation (Figure 6C). It has been reported that curcumin enhances *N*-acetylglucosaminyltransferase III (GnT-III) transcription, which transfers the bisecting GlcNAc to the core mannose of complex *N*-glycans [32] and inhibits an α6ß4 integrin dependent breast cancer cell motility and invasion [33], and interaction between α6ß4 integrin and EGFR [34]. Taken together, our results and above reports may indicate that increased bisecting GlcNAc on ß4 integrin decreased galectin-3 mediated EGFR-ß4 integrin complex formation, resulting in down-regulation of cellular signaling.

In conclusion, the present study is the first to clearly demonstrate that *N*-glycosylation ofß4 integrin can control its association with other molecules and its lipid raft localization on a membrane, and can regulate its dependent-cellular signaling and biological functions such as cell adhesion and migration. ß4 integrin is a key molecule in tumor formation and cancer metastasis, and the results of the present study suggest that the *N*-glycan on ß4 integrin is one of the determinants of cancer characteristics, which, therefore, suggests this could represent a new therapeutic target.

## Materials and Methods

### Antibodies and Reagents

The experiments were performed using the following antibodies (Abs): a rat mAb specific for integrin α6 (GoH3); mouse mAbs to integrin α3 (P1B5) and EGFR (528); rabbit pAbs to EGFR (1005), and integrin ß4 (H101) (Santa Cruz Biotechnology, Inc., Santa Cruz, CA); mouse mAbs to integrin ß1 (JB1A) and integrin ß4 (3E1) (Millipore); mouse mAbs to the human laminin ß3 chain (Kalinin B1), extracellular signal-regulated kinase1 (ERK1) (MK12), caveolin-1 (2297), plectin (31) and paxillin (349); a rat mAb to integrin ß4 (439-9B) (BD Transduction Laboratories, Lexington, KY); a sheep pAb to EGFR (Upstate, NY); a mouse mAb to phospho-ERK1/2 (E10); and, rabbit pAbs to Akt and phospho-Akt (Ser 473) from Cell Signaling Technology. A mouse mAb against integrin ß1 (P5D2) and a rat mAb against integrin α5 (BIIG2), which were obtained from Developmental Studies Hybridoma Bank, University of Iowa, were purified from the hybridoma supernatant using a protein A-Sepharose™ 4 Fast Flow column (GE Healthcare). The control rat, rabbit and mouse IgGs were obtained from Santa Cruz Biotechnology, Inc.; A rabbit mAbs to integrin α6 (EPR5578) and phospho-Akt (Ser 473) (EP2109Y) from EPITOMICS; Affinity purified rabbit anti-rat IgG from Vector Laboratories (Burlingame, CA); Purified human plasma fibronectin from Millipore; rat tail type I collagen from BD Transduction Laboratories; 3,3′-dithiobis[sulfosuccinimidylpropionate] (DTSSP) cross-linker from Thermo Scientific (Rockford, IL); and, Alexa Fluors 488 and 546 and Alexa Fluor 546-conjugated phalloidin were purchased from Invitrogen (Carlsbad, CA). Biotinylated DSA, L_4_-PHA, E_4_-PHA, ConA, SSA and MAM lectins were from Seikagaku Biobusiness Corporation (Tokyo, Japan). A mouse mAb against the human laminin γ2 chain (D4B5) and purified recombinant laminin-332 [35] were a generous gift from Dr. Kaoru Miyazaki (Yokohama City University, Yokohama, Japan). Purified human laminin-332 was prepared as described previously [31].

### Expression Vectors

The *N*-glycosylation site-defective mutant cDNA was obtained by PCR using specific primer sets and KOD Plus polymerase (TOYOBO). For the first PCR, WT ß4 integrin cDNA in pENTR1A vector (Invitrogen) was used as a template. The mutation was introduced into ß4 integrin cDNA one by one. The cDNA sequence was verified by sequencing at each step. The final construct was recombined from pENTR1A to the LZRS blast retroviral vector, including a Gateway cassette, using the LR clonase II Enzyme mix (Invitrogen) by a recombination reaction.

### Cell Culture

ß4 null keratinocytes, which were obtained from a patient with epidermolysis bullosa with pyloric atresia, and normal immortalized keratinocytes were a generous gift from Dr. M. Peter Marinkovich (Stanford University). These keratinocytes were grown in a 50/50 mixture of defined keratinocyte medium (Invitrogen) and medium 154 (Cascade Biologics, Portland, OR) containing penicillin and streptomycin sulfate. Modified human 293 phoenix cells were cultured in DMEM supplemented with 10% FCS, penicillin and streptomycin.

### Retrovirus Infection

Retrovirus vectors were transfected into 293 phoenix cells using FuGENE^®^6 transfection reagent (Roche, Germany). After transfection, cells were selected with 5 µg/ml puromycin; the retrovirus then was produced in 293 phoenix cells. One day before infection, 4×10^5^ keratinocytes were plated in 6-well plates. After incubation with 5 µg/ml polybrene (Sigma-Aldrich) for 15 minutes, keratinocyte media were exchanged to 10 ml retroviral supernatant and another 5 µg/ml polybrene was added. Plates were centrifuged at 2,100 rpm for 1 hour at 32°C using a Hitachi CF16RXII centrifuge machine. After centrifuge, the media were replaced by keratinocyte growth media and the cells were maintained. To establish a cell line, cells were selected with 5 µg/ml blasticidine S (Calbiochem).

### Preparation of Cell Lysates and Immunoprecipitation

Cell lysates were prepared as follows. The cells were washed twice with cold PBS and then lysed with a lysis buffer (1% Triton X-100, 20 mM Tris-HCl (pH 7.4), 150 mM NaCl, 5 mM EDTA) containing a protease inhibitor cocktail (Nacalai tesque, Japan) and a phosphatase inhibitor cocktail (Roche). After incubation for 10 min on ice, cell lysates were clarified by centrifugation at 15,000 rpm for 10 min at 4°C. The resultant supernatant was used in the following experiments. The protein concentration was determined using a protein assay kit (Nacalai tesque, Japan). For immunoprecipitation, protein G-Sepharose Fast Flow beads (Amersham) were added to the cell lysate, followed by rotation for 1 h at 4°C to remove nonspecific binding protein from the beads. After centrifugation, the primary Ab was added to the supernatant and rotated overnight at 4°C. Then, protein G-Sepharose was added and followed by 2 h incubation at 4°C. Immunoprecipitates were washed five times with STEN washing buffer (50 mM Tris-HCl (pH 7.5), 150 mM NaCl, 2 mM EDTA, 0.2% NP40 (v/v)), suspended in reducing sample buffer, and heated at 95°C for 5 min.

### Immunoblotting

For the immunoblotting analyses, proteins resolved by SDS-PAGE were transferred to nitrocellulose membranes. The blots were probed with each specific Ab or biotinylated lectins. Immunoreactive bands were detected using an ECL detection kit (GE Healthcare, UK), a SuperSignal West Dura Extended Duration Substrate kit (Thermo Scientific, IL) and a Vectastain ABC kit (Vector Laboratories, CA). The band intensity was calculated using NIH ImageJ software.

### Flow Cytometry Analysis

Cells were detached from a 10-cm dish using trypsin containing 1mM EDTA. After quenching trypsinization with medium containing 10% FCS, cells were washed twice with PBS containing 1 mM EDTA and incubated with primary Ab or control IgG for 30 min on ice, followed by incubation with the appropriate secondary Ab. After washing three times with PBS containing 1 mM EDTA, flow cytometric analysis was performed using CellQuest software with a FACSCalibur (BD Biosciences).

### Immunofluorescence Microscopy

A 200 µl aliquot of cell suspension (2 × 10^5^ cells/ml of growth medium) was added to each glass-bottom dish (Asahi techno glass, Japan). For hemidesmosome components, keratinocytes were cultured in HAM's F12 : DMEM (1∶3) containing 10% fetal calf serum, 0.4 µg/ml hydrocortisone and 10^−6^ M isoproterenol (both from Sigma). After incubation for 24 h, the cells were washed with PBS and then fixed with 4% (w/v) paraformaldehyde in PBS for 10 min. For permeabilization, the cells were treated with 0.5% (v/v) Triton X-100 in PBS for 10 min. The fixed cells were blocked with 2% BSA in PBS for 1 h before staining with appropriate primary and secondary antibodies. Fluorescence images were obtained using either a fluorescence microscope (Olympus, Tokyo) equipped with 100 × / 1.35 UPlan-Apochromat oil immersion objectives or a LSM510 confocal microscope (CarlZeiss).

### Cell Adhesion Assay

The cell adhesion assay was performed as described previously [15]. Briefly, each well of a 96-well ELISA plate (Costar, Cambridge, MA) was coated with laminin-332 and then blocked with 1% BSA. Cells (2 × 10^4^ cells) were added to each well in supplement-free keratinocyte growth medium and incubated for 20 min. After non-adherent cells were removed by vigorous shaking, adherent cells were fixed with 25% (w/v) glutaraldehyde for 10 min and stained with 0.5% crystal violet (w/v) in 20% (v/v) methanol for 10 min. The dye was extracted using 0.1 M sodium citrate in 50% methanol (v/v) for 30 min. Then, absorbance at 590 nm was measured using a microplate reader.

### Cell Migration Assay

A glass-bottom dish (Asahi techno glass, Japan) was precoated with 2 µg/ml laminin-332 and then blocked with 1% BSA for 1 h at 37°C. A 200 µl aliquot of keratinocyte suspension (4 × 10^4^ cells/ml) in growth medium was added to each laminin-332 precoated glass-bottom dish. After incubation for 1 h at 37°C to allow cells to adhere to the laminin-332, cell migration was monitored for 4 h using time-lapse video equipment (Carl Zeiss, Germany).

### Determining the Percent of Polarized Cells

Keratinocytes were fixed with paraformaldehyde and stained with anti-paxillin Ab and phalloidin. Fluorescence and phase-contrast images were taken of three separate fields containing approximately 100 cells for each culture plate. For a cell to be scored as polarized, it needed to possess all three defining properties of the fan cell morphology: (1) a lamellipodia that extends to the front of cells; (2) a nucleus polarized to the rear of the cell; and, (3) a paxillin that localizes to the front of the cell but not around the circumference of the cell. Cells that were spread, but did not fulfill these three parameters, were counted as nonpolarized.

### Covalent Cross-linking Experiment

Cells were washed with ice-cold PBS three times and added to a 2 mM DTSSP solution in PBS. After incubation for 2 h on ice for covalent cross-linking, 1M Tris-HCl (pH7.5) was added to a final concentration of 20 mM Tris, and the quenching reaction was incubated for 15 min on ice. The cells were then washed with PBS twice and lysed in a lysis buffer.

### Integrin Cross-linking

Cells were transferred to 60-mm dishes (2 × 10^6^ cells per dish) 18 h prior to the integrin clustering experiment. Immediately prior to integrin clustering, cells were washed twice with PBS. The cells in supplement-free keratinocyte growth medium were incubated on ice for 30 min in the presence of a 1∶50 dilution of the anti-ß4 integrin Ab. The cells were washed with supplement-free keratinocyte growth medium twice and then incubated for 10 min at 37°C in the presence of a 1∶100 dilution of rabbit anti-rat IgG (10 µg/ml) or in the absence of secondary Ab as a control. Following the incubation with the secondary Ab, the cells were immediately placed on ice and the medium was removed from the monolayer. The cells were washed with cold PBS twice and then lysed in a lysis buffer.

### Statistical Analysis

Comparisons between two groups were made using an unpaired Student's *t*-test, and among groups by one-way analysis of variance (ANOVA) followed by a Bonferroni post-test, with GraphPad Prism Version 5.0a software. A P value of 0.05 was taken as the threshold for statistical significance. The images shown are representative of at least three independent experiments performed.

## Supporting Information

Figure S1 **Analysis of single** ***N*** **-glycosylation mutants or mutant forms with a combination of these mutation sites expressing keratinocytes.** (A) Cell lysates from single *N*-glycosylation site mutants expressing ß4 integrin-deficient keratinocytes (ΔN^327^, ΔN^491^, ΔN^579^, ΔN^617^, ΔN^695^) as well as lac and WT keratinocytes were run on a 6% SDS-polyacrylamide gel and probed with an anti-ß4 integrin Ab. (B) Cell morphology of the indicated keratinocytes during cell culture. (C) Cell lysates from three mutant forms with a combination of these mutation sites (ΔN^327, 491, 579^, ΔN^617, 695^, ΔN^579, 617, 695^) expressing ß4 integrin-deficient keratinocytes as well as lac and WT keratinocytes were run on a 6% SDS-polyacrylamide gel and probed with an anti-ß4 integrin Ab. (D) Cell morphology of the indicated keratinocytes during cell culture.
(TIF)

Figure S2 **Lack of** ***N*** **-glycosylation on ß4 integrin does not affect laminin-332 secretion and deposition.** (A) Conditioned medium (CM) and deposited matrix (ECM) from WT or ΔN keratinocytes were prepared as described previously (Kariya Y. et al., (2004) *J. Biol. Chem.* 279: 24774-24784.) and were run on a 6% SDS-polyacrylamide gel and probed with an anti-laminin ß3 mAb. (B) WT or ΔN keratinocytes were cultured for 24 h, and stained with an anti-laminin-γ2 mAb to visualize the deposited laminin-332 under the cells. (C) The hemidesmosome structure of WT or ΔN keratinocytes. Keratinocytes were cultured in HAM's F12 : DMEM (1∶3) containing 10% fetal calf serum, 0.4 µg/ml hydrocortisone and 10^−6^ M isoproterenol (both from Sigma). After 24 h, cells were fixed by 4% paraformaldehyde and then permeabilized with 0.5% Triton X-100 at room temperature for 15 min, followed by staining with the indicated Abs.
(TIF)
