# Supplementary figures and images for: N-Glycosylation of ß4 Integrin Controls the Adhesion and Motility of Keratinocytes
Source: PLoS One. 2011 Nov 2;6(11):e27084. doi: 10.1371/journal.pone.0027084 (PMC3206902; doi:10.1371/journal.pone.0027084)

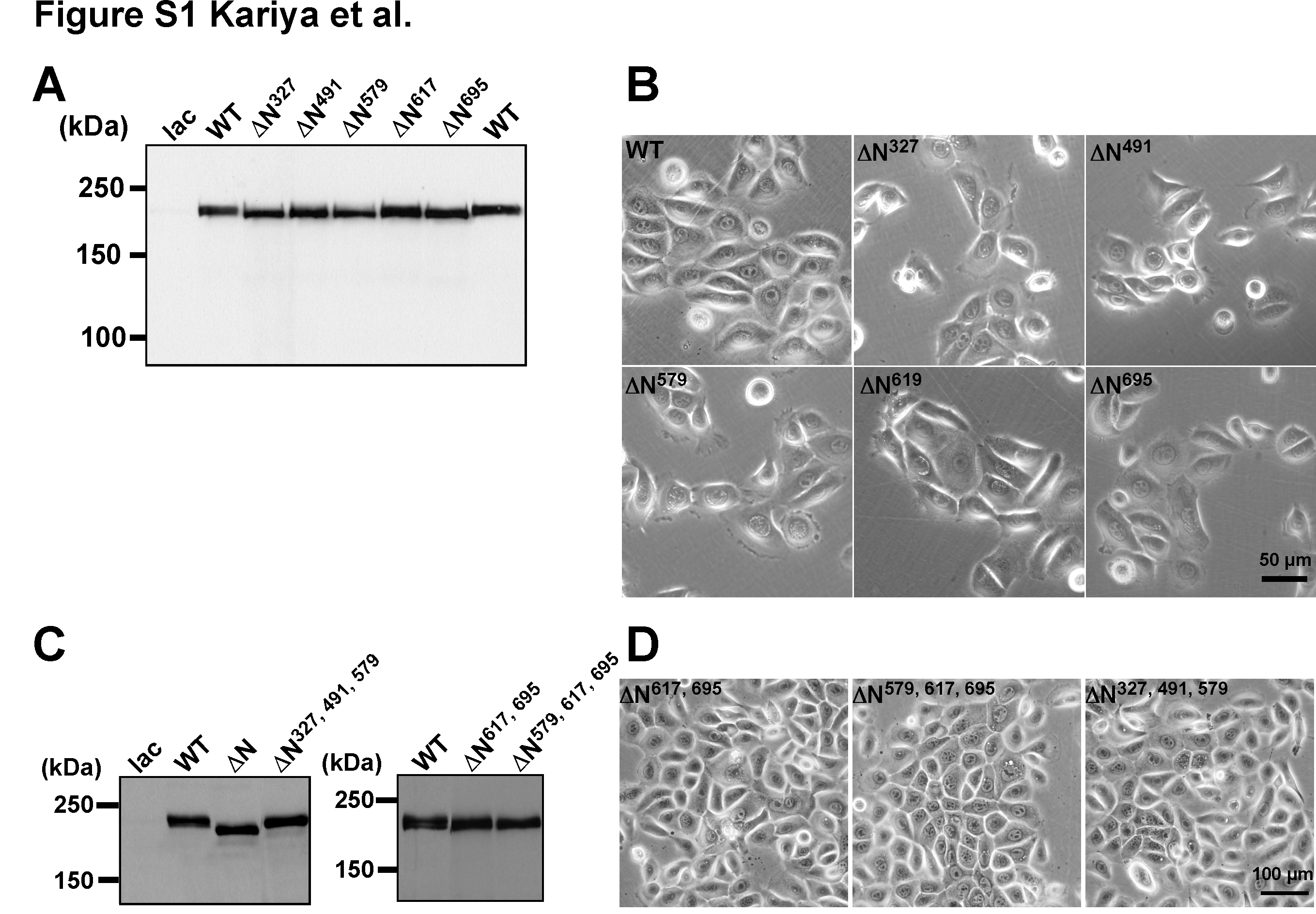

Supplement: Figure S1 — Analysis of single N -glycosylation mutants or mutant forms with a combination of these mutation sites expressing keratinocytes. (A) Cell lysates from single N-glycosylation site mutants expressing ß4 integrin-deficient keratinocytes (ΔN327, ΔN491, ΔN579, ΔN617, ΔN695) as well as lac and WT keratinocytes were run on a 6% SDS-polyacrylamide gel and probed with an anti-ß4 integrin Ab. (B) Cell morphology of the indicated keratinocytes during cell culture. (C) Cell lysates from three mutant forms with a combination of these mutation sites (ΔN327, 491, 579, ΔN617, 695, ΔN579, 617, 695) expressing ß4 integrin-deficient keratinocytes as well as lac and WT keratinocytes were run on a 6% SDS-polyacrylamide gel and probed with an anti-ß4 integrin Ab. (D) Cell morphology of the indicated keratinocytes during cell culture. (TIF) [file pone.0027084.s001.tif]

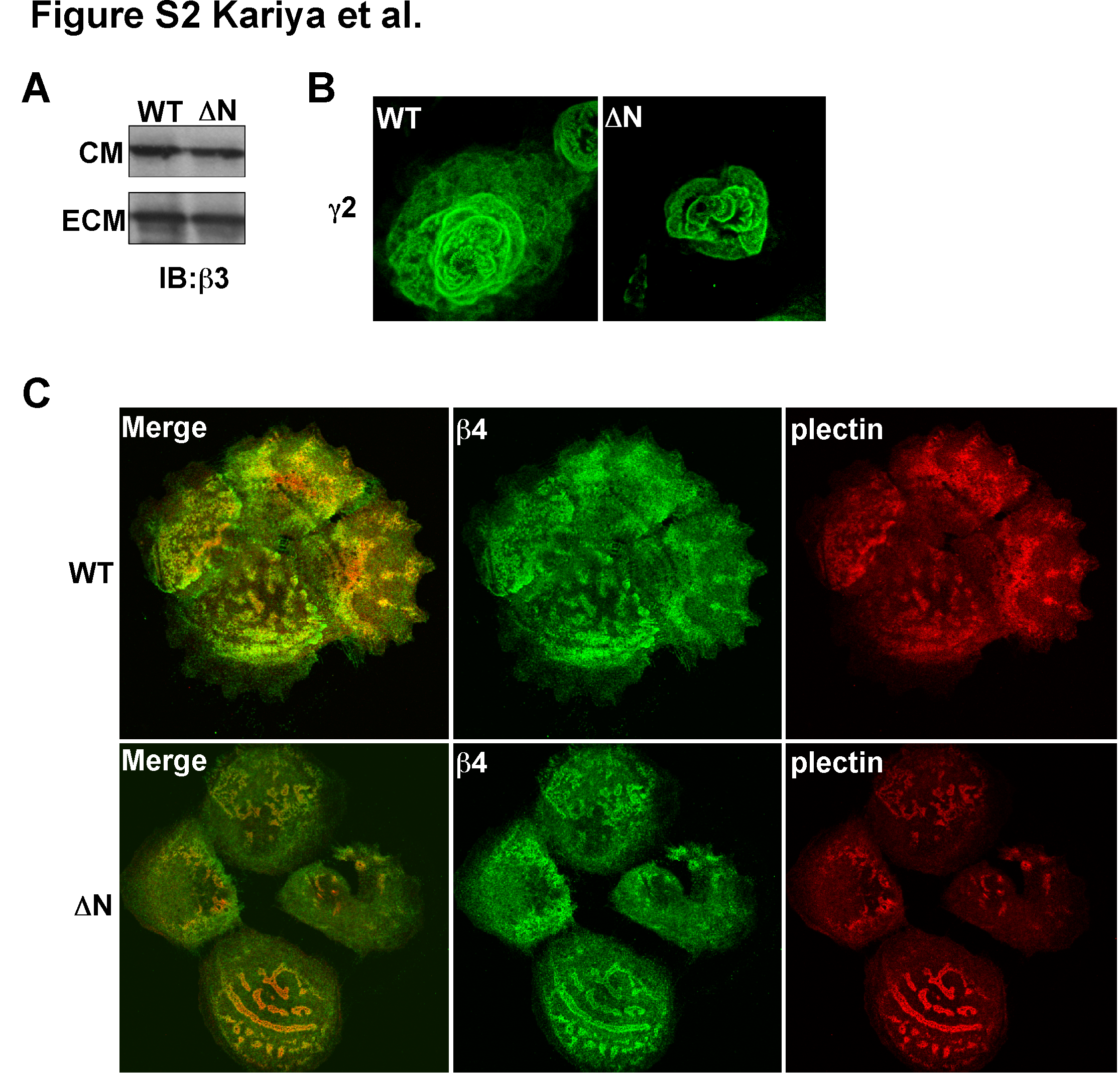

Supplement: Figure S2 — Lack of N -glycosylation on ß4 integrin does not affect laminin-332 secretion and deposition. (A) Conditioned medium (CM) and deposited matrix (ECM) from WT or ΔN keratinocytes were prepared as described previously (Kariya Y. et al., (2004) J. Biol. Chem. 279: 24774-24784.) and were run on a 6% SDS-polyacrylamide gel and probed with an anti-laminin ß3 mAb. (B) WT or ΔN keratinocytes were cultured for 24 h, and stained with an anti-laminin-γ2 mAb to visualize the deposited laminin-332 under the cells. (C) The hemidesmosome structure of WT or ΔN keratinocytes. Keratinocytes were cultured in HAM's F12 : DMEM (1∶3) containing 10% fetal calf serum, 0.4 µg/ml hydrocortisone and 10−6 M isoproterenol (both from Sigma). After 24 h, cells were fixed by 4% paraformaldehyde and then permeabilized with 0.5% Triton X-100 at room temperature for 15 min, followed by staining with the indicated Abs. (TIF) [file pone.0027084.s002.tif]
